# Motivation for solitude: a validation of the Czech version of the MSS-SF instrument

**DOI:** 10.1192/j.eurpsy.2026.11692

**Published:** 2026-07-31

**Authors:** K. Knížková, N. Doubková, V. Juríčková, R. Heissler, M. Preiss, P. Mohr

**Affiliations:** 1 National Institute of Mental Health, Klecany; 2 Department of Psychiatry, First Faculty of Medicine, Charles University and General University Hospital in Prague, Prague; 3 Center for Advanced Studies of Brain and Consciousness, National Institute of Mental Health, Klecany; 4 AMBIS University; 5 School of Psychology, University of New York in Prague, Prague, Czech Republic

## Abstract

**Introduction:**

Positive solitude appears to have a positive impact on mental health, social relations, greater life satisfaction, and creativity. Contrastingly, loneliness and social isolation often seem to be associated with depressive symptoms, and an increased risk of cardiovascular disease, stroke, cognitive decline or suicide. The “Motivations for Solitude Scale - short form” (MSS-SF) asks the question of “why” individuals seek solitude, such as self-discovery, social disinterest or autonomy, as it distinguishes between self-determined and non-self-determined solitude.

**Objectives:**

The purpose of this study was to validate the MSS-SF questionnaire in Czech adults and to assess its psychometric properties. As the instrument was originally validated in adolescents and emerging adults, we compared the analyses between the adult (26 to 64 years old) and the emerging adult (18 to 25) subgroups. We also tested for convergent validity with relevant measures.

**Methods:**

The final sample consisted of 1274 adults between 18 and 64 years old (M = 37.4, SD = 12.1). The measures used in the study were MSS-SF, Positive Solitude Scale, the UCLA Loneliness Scale and DeJong Gierveld Loneliness Scale. Reliability was assessed using Cronbach’s alpha. To test the validity, we computed an inter-item correlation as well as correlations with the other questionnaires. For factorial validity, we run a confirmatory factor analysis. To compare the MSS-SF in emerging adults vs. adults, we used a multigroup factor analysis and independent samples t-tests.

**Results:**

The two-factor model of the MSS-SF demonstrated a good fit to the data with all indices within the acceptability range. The correlation of the items supported the two subscales design of the questionnaire. The subscales correlated with other instruments suggesting a good concurrent validity. Both self-determined solitude and non-self-determined solitude differed significantly between the groups. A multigroup analysis showed that the two-factor model provided a good fit to the data in both groups, with significant loadings for all items.

**Conclusions:**

The Czech version of the MSS-SF is a valid instrument to measure motivation for solitude in adults as well as emerging adults. The two-subscale solution fit the data and correlated with other measures of loneliness and solitude. In comparison with adults, emerging adults scored lower on self-determined solitude and showed similar results across the loneliness instruments.

**Disclosure of Interest:**

None Declared

